# “Anxiety or enjoyment, I feel pleasant to welcome them both”: thematic analysis of a Chinese PhD student’s personal growth experiences

**DOI:** 10.3389/fpsyg.2023.1173734

**Published:** 2023-08-31

**Authors:** Hua Chen

**Affiliations:** School of International Education, Shandong University of Finance and Economics, Jinan, China

**Keywords:** anxiety, enjoyment, personal growth, well-being, research diaries, autoethnography

## Abstract

Engaging with research is an emotionally demanding experience and a trajectory full of difficulties, challenges, and stress. This autoethnographic study explored my personal experiences as a PhD student in a four-year program and conducted a qualitative thematic analysis by analyzing 550 research diary entries collected between September 2018 and June 2022, in which supervisor feedback and reviewer comments were part of the content. Three recurring, unique, and salient themes pertaining to my personal experiences were identified: being fraught with anxiety, gaining a sense of enjoyment, and achieving personal growth. Whereas anxiety was from publication and dissertation writing, foreign language writing, and individual stressors, enjoyment was gained from the support network and conducting research. My personal growth was reflected from sustained engagement and improved autonomy. In the process, I experienced some negative emotions, but found more enjoyment. The findings indicate that anxiety and enjoyment are fluctuating, co-occurring, and reciprocal. The findings call for more attention to the role of research diary writing in scaffolding PhD research, providing emotional support, and facilitating personal growth and well-being of PhD students.

## 1. Introduction

PhD students’ well-being is crucial for higher education (e.g., Schmidt and Umans, 2014; Schmidt and Hansson, 2018), as well-being might affect students’ research and teaching productivity, research policy, and higher education quality (e.g., Levecque et al., 2017; Schmidt and Hansson, 2018). However, the majority of PhD students experience poor well-being (e.g., Langford, 2010; Beasy et al., 2021). They encounter difficulties and challenges (e.g., Park, 2005) and experience struggles and stress (e.g., Barry et al., 2018; Liu and Abliz, 2019; Huang, 2020). The unpredictability and non-linearity of progress during the PhD might bring varied and mixed emotional changes (Juniper et al., 2012). Students face challenges and might experience some negative emotions, such as depression, anxiety, and anguish. The struggles against these negative emotions might result in the decrease in confidence, amplification of frustration, and a higher level of anxiety. Such frustrations and anxiety might bring negative influences on their PhD trajectory and well-being. Given the role of PhD students’ personal growth and well-being, it is of critical importance to find a way to help them gain confidence in research engagement and manage unpleasant emotions.

Although current research has primarily focused on the importance of emotion for PhD students, little is known about what experiences, particularly emotional experiences PhD students might have during the entire PhD studies, and how they face up with the challenges to enhance their well-being in Chinese context. Studies have shown that emotion plays an important role in the doctoral experiences (Cotterall, 2013), and stress harms students’ health (Russell-Pinson and Harris, 2019). PhD students’ research and emotional experiences have been investigated in some countries, such as Japan (e.g., Casanave, 2010), Sweden (e.g., Langum and Sullivan, 2017), U.S. (e.g., Russell-Pinson and Harris, 2019), and China (e.g., Wang et al., 2019; Geng and Yu, 2022). These studies have provided useful insights into the understanding of PhD students’ experiences. However, research diaries of PhD students, as an important scaffolding for students (Engin, 2011) and a cathartic tool (Browne, 2013), have not received sufficient attention from scholars. This study, situated in a Chinese context, used an autoethnographic approach to explore my personal experiences as a PhD student through analyzing the research diary entries across 4 years to reveal the personal growth.

## 2. Literature review

### 2.1. Studies on diaries

Diary is defined as “a document created by an individual who has maintained a regular, personal and contemporaneous record” (Alaszewski, 2006, p.1). Diaries record “events, experiences, thoughts and feelings” of diarists (e.g., Alexander et al., 2016, p. 19) and provide a rich and valuable data source about diarists’ personal experiences (e.g., Alaszewski, 2006; Allen, 2013). It serves the function of healing effects for diarists (Glass et al., 2019). Diaries have been used to investigate teachers’ beliefs (e.g., Carson and Longhini, 2002; Allen, 2013) and students’ language learning (e.g., Bailey, 1983, 1990; Bhattacharya and Chauhan, 2010; Shelton-Strong and Mynard, 2020). The studies showed that diaries helped teachers record their experiences (Allen, 2013), facilitated the autonomy augmentation (Bhattacharya and Chauhan, 2010), and promoted students’ positive feelings and motivation in language learning (Shelton-Strong and Mynard, 2020).

Research diaries have been used to document the researchers’ reflections (e.g., Silverman, 2005; Gibbs, 2007). A research diary may include the research process (e.g., Borg, 2001), methodological steps (e.g., Browne, 2013), and researchers’ knowledge development (e.g., Engin, 2011). Several studies have explored the functions of research diaries, such as a tool for scaffolding (Engin, 2011) and a cathartic tool (Browne, 2013). These studies focused on the role of diary writing for PhD students in different contexts, and used researchers’ personal diary entries to describe the PhD work. Engin (2011) investigated the role of research diaries in helping students learn about research and how to be a researcher from the socio-cultural theory of learning. Browne (2013) explored the benefits of writing and maintaining research diaries for recording the emotional and practical challenges of fieldwork in unfamiliar settings. Ridgway (2022) focused on the interconnections between memorable events and junctures with her doctoral journey. She used three diary entries in the depths of her PhD to reveal the role of writing diaries for “identifying the echoes between personal and graduate school experiences” (p. 1). These studies have paved a way for using research diaries among PhD students and call for more attention to the use of research diaries to probe into PhD students’ experiences in different disciplines and contexts.

### 2.2. Studies on PhD students’ experiences

Research into PhD students’ experiences has focused on both research experiences and emotional experiences. As writing is “central to the process of developing a scholarly identity and fundamental to the doctoral experience” (De Magalhaes et al., 2019, p. 4), studies of PhD research experiences have investigated writing related experiences, such as writing styles (e.g., Casanave, 2010), writing expertise (e.g., Casanave, 2019), writer identity (e.g., De Magalhaes et al., 2019) and related challenges and emotions (e.g., Badenhorst, 2018; Beasy et al., 2021). Casanave (2010) illustrated how three PhD students developed their writing styles over a two-year period and emphasized the importance of PhD students’ dissertation writing experiences. Langum and Sullivan (2017) investigated the challenges that non-native English PhD students faced in the routes into their international academic English publications. The study reported that hesitancy and distance were common among Second Language (L2) doctoral researchers. They experienced feelings of insecurity in communicating research results and translating their ideas and primary thoughts into English, and remoteness in the development of bi-literate academic writers. In the study of Badenhorst (2018), students experienced writing anxiety, especially when receiving negative feedback on their dissertations or papers for publication. The study stressed the emotional nature of writing for graduate students and presented three pedagogical strategies: free-writing, negotiating negative internal dialog, and using objects to externalize feelings. With these strategies, students could better recognize their emotions, make decisions, and develop agency. De Magalhaes et al. (2019) investigated two English as an Additional Language (EAL) PhD students’ challenges associated with developing scholarly identities during their candidature. It reported students’ vulnerability in trying to adjust their voices to meet the norms. Casanave (2019) pointed out that raising PhD students’ awareness of accepting the performance and considering it to be a part of an academic life, might “help prevent debilitating anxiety that comes from expectations that are set unrealistically high” (p. 43). Investigating into academic research among PhD students opened up space for insights into their personal growth and well-being.

Existing studies have also shown that PhD students experience intense emotions apart from conducting research (Bettinson and Haven-Tang, 2021). PhD students’ emotional experience studies have explored a set of emotions, particularly the negative emotions, such as anxiety, frustration, and anguish (e.g., Aitchison et al., 2012; Cotterall, 2013; Russell-Pinson and Harris, 2019; Geng and Yu, 2022). Casanave (2014) pointed out that emotion was a part of PhD students’ dissertation journey. Emotions provided motivational energy for students to persist until graduation (McCormack, 2009). Previous studies suggested that PhD students be supported to learn skills of attending to the stress to maintain and enhance their well-being (e.g., Russell-Pinson and Harris, 2019). Given the challenges of academic research faced by PhD students and the complexity of emotional changes experienced by them, more studies of PhD personal experiences are required to reveal a full picture of their personal growth in both research and emotional experiences in various contexts.

### 2.3. Studies on PhD students’ well-being

Well-being refers to “the individual’s experience of his or her health” (Medin and Alexanderson, 2001, p. 75). PhD students’ well-being has been termed as internal reflection (Haynes et al., 2012), being true to oneself (Schmidt and Umans, 2014), and self-care (Kumar and Cavallaro, 2018). Well-being can be reflected from the positive relationship with others, personal growth, environmental autonomy, autonomy, purpose in life, and self-acceptance (Ryff, 1989).

PhD students’ well-being has been related to many factors, such as productivity and efficiency, research policy, problem-coping strategies, support network, the culture, and the consideration of well-being by students themselves (e.g., Levecque et al., 2017; Schmidt and Hansson, 2018; Jackman et al., 2022). Levecque et al. (2017) assessed the prevalence of PhD students’ mental health problems among 3,659 students in Belgium. Results of the study showed that 32% of students were at a risk of having or developing psychiatric disorder, particularly depression. Jackman et al. (2022) investigated the early stages of PhD students’ doctoral research and stressed the importance of self-care for PhD students. The study provided some common self-care strategies (i.e., physical activity, hobbies, and rest periods) and suggested that PhD students be educated on some self-care practices, such as physical activity and rest periods. Such suggestions have provided useful implications for the intervention strategies to enhance PhD students’ well-being in the early stages of their study. Based on these studies, how to enhance PhD students’ well-being during the entire studies remains to be explored.

Overall, most studies have limited their investigation of PhD students’ experiences through researchers’ perspective. Given the complexity of PhD students’ research experiences and the variety of emotions experienced by PhD students, their personal experiences could be manifested in a nuanced way. Previous studies have centered more around some anxiety-filled experiences. It remains unclear what other emotions PhD students might experience, what sources might cause the emotions, and how they might enhance their well-being. Furthermore, although several studies have explored the usefulness of research diaries in their disciplines and contexts, a longitudinal research diary study in English as a Foreign Language (EFL) field and in the context of mainland China covering the entire PhD study is scarce. To address these research gaps, the present study aims to explore my personal experiences as a PhD student in a four-year program through analyzing chronologically organized data in the context of China.

## 3. Methods

### 3.1. Research context

I enrolled in a Foreign Linguistics and Applied Linguistics PhD program at one of the key universities in the north of China in fall 2018. The program length was from 4 to 6 years. I need to meet the graduation requirements to get a PhD degree, including courses, publications, and a dissertation. I worked full-time at another university in the same city, where I studied as a PhD student. I was a little bit anxious before I embarked on the journey, as I knew getting a PhD degree would be a difficult and challenging task. I worked hard to complete the compulsory and selective courses (most in English and some in Chinese) and started early on writing academic journal articles. However, the challenges of selecting research topics, writing and revising manuscripts, publishing research articles, and completing a dissertation, were more challenging than I had previously thought.

The major challenges were threefold. The first challenge was to publish innovative academic articles on SSCI-indexed or CSSCI-indexed journals. The second challenge was to conduct an appropriate research project and complete a PhD dissertation. The third challenge arose from my expectations of transforming from a novice researcher to a PhD graduate who could construct a desired academic identity. After a careful self-assessment, I started writing research diary entries to record my personal experiences. At the beginning of my first year, the research diaries focused more on the justification of the research design and details of the research development, difficulties and challenges I experienced in learning to conduct research, and corresponding strategies I employed. However, with more difficulties from academic writing, submission, and rejection by journals, I could hardly control my emotions. It was in this situation that I wrote more about my emotions and emotion regulation strategies. Through writing about my emotions, especially the mixed negative emotions going forth and back, I found that writing research diaries embodied the dual role of being an emotional outlet and an emotional support. It helped to document and reflect on my research and emotional experiences, and calmed me down. Realizing the mediating role of writing research diaries in the co-development of my research competence and emotional management, I took this activity as an important task and wrote diaries on a regular basis. Therefore, the diaries could present a clear overall picture of my research trajectory and emotional changes in the 4 years’ PhD program.

### 3.2. Data collection and analysis

This study adopted autoethnography to explore my personal experiences as a PhD student, especially the emotional experiences across 4 years. This approach has the advantage of calling up personal embodied experiences (Maslen, 2022), helping to observe and analyze their own emotions (Buckley, 2015), offering an insider perspective, and revealing the meaning of lived experiences (Ellis et al., 2011). It also has the advantage of promoting personal well-being (Ellis, 2013) through recounting personal experiences (Lapadat, 2017) and emotional experiences (Akehurst and Scott, 2021).

Diaries were the data source, and supervisor feedback and reviewer comments were part of the content of the diary entries. I started writing Chinese diary entries from the first semester, as writing in my mother language was an easier way for me to narrate personal experiences, particularly the complicated and varied emotions. I wrote 35 research diary entries in this semester. In the dairy entries, I quoted supervisor feedback and reviewer comments in the original languages (i.e., English or Chinese). With more stress from course assignments and publication demands, I embarked on a journey of writing about two research diaries per week. In all, I collected 550 research diary entries with the total length of 271,903 words written between September 2018 and June 2022. The diary entries were based upon my personal experiences and interaction with other people, such as my supervisor, reviewers, friends, and family members.

Qualitative thematic analysis was used to help identify, analyze, and report the major themes and sub-themes (Braun and Clarke, 2006; Yin, 2011). Thematic analysis could summarize the key features of the data (Nowell et al., 2017) and generate unanticipated insights from the data (Braun and Clarke, 2006). Qualitative research software NVivo12 was employed to help analyze the diary entries. I followed the coding scheme: (1) familiarizing myself with all diary entries; (2) generalizing the initial codes from the diary entries; (3) identifying the common themes used to present my experiences, especially emotional experiences; (4) reviewing all the themes; (5) defining and naming three major themes; and (6) producing the report of my PhD personal experiences. Three recurring, unique, and salient themes pertaining to my personal experiences during the 4 years’ PhD program were identified: being fraught with anxiety, gaining a sense of enjoyment, and achieving personal growth.

### 3.3. Rigor of the study

I used the criteria of credibility, transferability, dependability, and confirmability (Lincoln and Guba, 1985) to ensure the rigor of the study. Credibility was guaranteed through the prolonged engagement with the data. As this autoethnographic study did not have other participants or co-authors, I invited a researcher colleague, who had over 30 years’ teaching and research experiences at the university where I worked, to help verify that the diary entries reflected my personal experiences. I shared with and asked her to give comments on some diary entries. For example, she helped with assessing whether some diary entries describing my regular work (teaching) in workplace, my EFL writing experiences, and social support I received, were the true description of my experience. To increase the credibility, a researcher colleague was invited to provide an external check. Transferability was guaranteed by the transparency of the data, the description of the research context, data collection, coding, and formulating themes and sub-themes. Dependability was enhanced by a detailed description of data analysis. Confirmability was established by coding the data. There were ongoing discussions with my researcher colleague in terms of the preliminary findings, emerging codes, sub-themes, and themes. Inter-rater reliability was established with the same researcher colleague to study and code 25% of the data. This produced an inter-rater reliability of 0.88.

## 4. Findings

Looking back on my 4 years’ experiences as a PhD student, I experienced anxiety mostly in the preliminary stages, and anxiety waxed and waned. I regarded this kind of emotion advantageous to increase attentiveness to academic research. Emotional self-support through writing research diaries and social support helped me find an effective way of gradually lessening and relieving anxiety. I gradually gained a sense of enjoyment. The reflection and reconciliation helped me develop myself in an all-round way, and in this process, I learned to take a more self-distanced perspective to evaluate myself and viewed these experiences as life path and predictors of well-being.

### 4.1. Being fraught with anxiety

The excitement at the news of admission to further my study as a PhD student after working some years was soon replayed by anxiety when I came to realize how challenging and difficult the PhD trajectory would be for the upcoming 4 or even 6 years. My anxiety mainly came from publication and dissertation writing, foreign language writing, and individual stressors. Such anxiety lasted mostly in the first 2 years and gradually lessened with the improvement of EFL writing skills and better perceptions of my identity as a novice researcher in the field.

#### 4.1.1. Anxiety from publication and dissertation writing

Choosing an appropriate research topic was the first cause of anxiety. Anxiety accompanied me from embarking on the first research topic to new ones. This source of anxiety was more obvious for me when I had to work on a new research topic. I noted down my anxious feelings in one diary entry.

*I found myself quite confused about what research topic I should focus on and what PhD dissertation project I should conduct. The more I thought about this, the more anxious I became. I have been reading a lot of research articles, but still I do not have a clear idea about how to narrow down a research topic and design my own research based on and informed by previous studies and theories. I am somewhat lost and unclear about what I could do in this field, and I seem to remain an onlooker when reading academic papers* (Diary, 18 April 2019).

Writing research articles for possible publications aroused anxiety. I felt anxious in every step of such type of writing, especially in the first year of PhD. Anxiety at this stage was mainly caused by the unfamiliarity with academic writing and inaccurate perceptions of myself as a novice researcher. The anxiety was elevated when I realized that all my submitted articles were not in the first round of peer review. For instance, after discussing with my supervisor whether we should email the publishing house for further update information, I wrote a diary entry:

*3:30 a.m. in the early morning. It is still dark. Quite unconsciously the idea occurred to me that I did not spend sufficient time writing research papers, bringing about some kind of negative emotions of anxiety and anguish spontaneously. The more I thought of this, the more overwhelmed I became. I have a lot of repressed feelings… The fear of publication failure is similar to that when I was not sure whether I could be enrolled in a desired university. I feel worried, as up till now, all my submitted articles have not been into the first round of peer review. So bad feelings* (Diary, 22 May 2019).

Finding an appropriate journal increased my anxiety, especially when I did not have confidence or felt inexperienced in academic research. This was more evident when one manuscript was rejected. The following diary entry described how I felt about finding a journal to submit my manuscript to.

*Time passes quickly. It has been almost one year since I enrolled in the PhD program. I find it quite difficult and challenging to find an appropriate journal, which might accept my first English manuscript. I do not have any confidence right now. What I have been doing is to submit one manuscript to another different journal if it is rejected. I feel so sad that my manuscripts have been rejected several times. The experiences made me feel more depressed every time I thought of these discouraging moments. I have no idea what I should do now, and perhaps I need to spend more time in finding more appropriate journals. It is such a difficult and challenging trajectory for a first-year PhD student* (Diary, 16 May 2019).

Waiting for the decision of journals caused substantial anxiety for me. This kind of anxiety intensified each time when the manuscript was rejected, and was more elevated when the revised version got rejected more than once. Checking the status of the submitted manuscripts was a daily routine for me. I tried to note down my anxiety and worries in one diary entry.

*4:00 a.m. in the early morning. The first thing I do after getting up is to check the status of the submitted articles. This has become a daily routine. Publication has been torturing me a lot. The more anxious I feel, the worse result it seems to have. I quivered a little bit when learning that one manuscript was rejected a moment ago. How I wish this were not true. Tears rolling down my cheeks, I could not control my feelings. I have made every possible effort to revise the manuscript. Now the more I think about it, the worse feelings I have. I become worried about another manuscript, as it might be rejected as well. This made me get a little bit desperate when I realize that the manuscript has been under review for ages…* (Diary, 15 May 2019).

Receiving feedback from my supervisor and comments from reviewers caused anxiety. The supervisor has been an authoritative figure in academia and set a good model for me. I took my supervisor’s feedback and suggestions carefully and implemented the feedback, but the feedback sometimes aroused a kind of anxiety for me. It was mainly caused by my EFL writing ability and perceptions of my research competence. When I received supervisor feedback, such as “? expression,” “? The subject of this sentence is different from that of the previous sentence. Please revise this sentence. It is a grammatical error here.” (Supervisor feedback, 2 March 2021), and “which implication comes first?” (Supervisor feedback, 5 June 2021), I had anxiety, even though I knew that the use of interrogative and imperative sentences lessened my anxiety to some extent, and I could improve my writing competence through making more effort.

I also experienced anxiety when receiving reviewers’ comments on manuscripts. Such anxiety lasted long and came back and forth. This kind of emotion was more obvious in the past one and half years of my PhD. Anxiety came to me when I read “The theoretical background of the paper is inadequate,” (Reviewer comments, 5 September 2019) and “There are sections of the paper which are confusing and should be reformulated,” (Reviewer comments, 21 December 2020). I noted my anxiety in one diary entry.

*I had mixed emotions while I was reading supervisor feedback…I experienced some kind of anxiety. I could not help pondering over how I could improve my EFL writing competence and better manage my emotions. Reading the feedback, I know that more improvement is needed…But, the difficulties seem to have put me into more anxiety…My emotions changed repeatedly from excitement and relief after submitting the manuscripts to anxiety and depression after receiving reviewer comments. The comments are constructive, but I still experience anxiety. These emotions have been with me every single day in the past one and half years. How I hope that one day there will be less anxiety and more enjoyment* (Diary, 29 December 2020).

#### 4.1.2. Foreign language writing anxiety

Foreign language writing anxiety was caused by unfamiliarity with the language, setting up appropriate arguments, and finding more authorized resources to support arguments. The gap between my foreign language proficiency and the required EFL writing competence has caused long-lasting anxiety. This source of anxiety ran across the draft and manuscript writing, revision, and submission. I could hardly get rid of the anxiety. It lessened gradually after I had more EFL writing experiences and self-care strategies. The following diary entry recorded this source of anxiety.

*A bad day today. One manuscript was rejected…I felt quite overwhelmed, but I knew that I had to accept it. I still have a lot of difficulties with EFL writing. There are other difficulties as well, such as subject-verb agreement, redundancy, repetition, and overuse of linking words. It is still a long way to go in foreign language writing* (Diary, 4 September 2019).

Finding authorized resources presented anxiety for me, especially in the first manuscript. I felt anxious when I failed finding more authorized resources and even more anxious when the reviewers suggested that I add more authorized resources. I wrote about this experience in my diary.

*Feeling a little bit frustrated today. It is a revision day. I added more authorized resources. It is a really difficult and challenging task. How embarrassed I felt when I did not find proper authorized resources to set up arguments. I need to read more related research articles, select appropriate materials, and use these articles to address the issue. Oh, what a day!* (Diary, 5 May 2020).

#### 4.1.3. Anxiety from individual stressors

The individual stressors came from work (teaching at one university) and family, which triggered my anxiety from time to time, as I worked full time and had a family to take care. I was well aware that obtaining a PhD degree was more challenging for a full-time teacher and mother, and I tried to balance among research, work, and family. Still, I was haunted by anxiety once I failed doing research as scheduled, and this kind of anxiety intensified, especially when I did not find any time doing research in a week or when one manuscript was rejected. Noted in many diary entries were cases of anxiety from these stressors: “Today I have to complete a 2,000-word work report. I feel a little bit anxious when I realize I do not have time working on papers,” (26 May 2020), “I feel so overwhelmed. I did not have any time on papers. Several days have passed,” (18 November, 2020), and “Today it is weekend, and I spent a day with family. I did not do any academic work, and I did not even make any improvements in my papers. I feel anxious.” (26 November 2020).

### 4.2. Gaining a sense of enjoyment

The enjoyment came primarily from the support network and conducting research. Such enjoyment provided an important opportunity for me to manage my complicated and varied emotions. The support from different sources was encouragement to me, whereas conducting research offered me opportunities to evaluate my academic writing performance and helped me develop positive appraisals of anxiety, which both led to better enjoyment.

#### 4.2.1. Enjoyment from the support network

The support network included self-support through writing research diary entries and social support through receiving positive comments from various sources. Writing diaries offered me an opportunity to reflect on past research and emotional experiences. Self-reflection improved my ability to activate emotions and cognition. I could better engage in assessing my academic writing performance. In doing so, I regarded diary writing as an emotional outlet, whereas social support reinforced my research engagement. The diary entry described how I valued this kind of writing.

*3: 45 a.m. in the early morning. I happened to read an online blog diary entry. The writer wrote that keeping diaries helped her record personal experiences, and writing had the healing power for her, especially when she felt at a loss or sought some kind of support. I was impressed by what she wrote in her diary, as we had similar feelings…Once I had no idea about how to write or revise the manuscripts, I wrote diaries and read previous ones to help me relieve the anxiety and calm down. In such a way, my anxiety got relieved to a large extent. I came to realize the importance of the progress I made and gained increasing confidence in myself by means of keeping diaries, which further motivated me to make greater effort to conduct research. More importantly, I have developed a stronger research interest and an increasing awareness of studying myself in the future* (Diary, 31 May 2020).

Social support I received included the positive comments and encouragements from my supervisor, reviewers, colleagues, family members, and friends. Their support brought me some sense of enjoyment. The constructive and positive feedback from the supervisor and reviewers were promoters for my development from anxiety to enjoyment and personal growth. The stressors from work and family were important social support sources facilitating my personal growth. I wrote about how one friend’s support helped me treasure my life as a PhD student. I noted that “One friend gave me very useful advice on topic sentences, logic, and structures within paragraphs. She has always been there whenever I feel worried and anxious. Such friendship is a treasure in my life, especially during my PhD,” (Diary, 3 December 2020).

The balance among teaching, research, and family helped me gain more enjoyment. I noted in the first year that “I have to balance among teaching, research, and family. Spending time with family is relaxation and gives me encouragements…I could get some useful feedback from the ongoing discussions with my colleagues,” (Diary, 19 March 2019). In the third year, I wrote that “I have well learned to balance among teaching, research, and family. I could gradually get used to the way how I reconcile with myself and find a way to release my emotions. Writing diaries benefits me, especially when I am experiencing a lot of stressors,” (Diary, 19 December 2021).

#### 4.2.2. Enjoyment from conducting research

My enjoyment from conducting research covered two stages of manuscript writing, i.e., revision and acceptance of manuscripts. Revision of manuscripts was the first most important enjoyment source for me. During the revision process, my enjoyment came from revision and the progress I made after rounds of revision. I noted down my enjoyment in one diary entry.

*A new day starts. I completed making revisions and was ready to submit the revised version to the journal in a minute. I felt more relieved and confident when I read through the response. I clicked the ‘submit’ button and took a deep breath. Although I was not very sure whether the revised manuscript would be accepted, I had a sense of enjoyment in revising, as I moved a step forward with persistence and diligence. Even though it might be rejected, I would not feel regretful as long as I have tried. In the past two months, I responded to the reviewer comments and revised the version repeatedly. It is not exaggerating to use the word ‘repeatedly’, as each time I was about to submit, I always identified some new errors in the manuscript, in which case, I had to revise it all over again. It is in the constant revision process that I became a better EFL writer with more interest in research* (Diary, 24 February 2021).

Acceptance of research articles brought me a great sense of enjoyment in academic research. Such enjoyment was an encouragement and a stress reliever for me to build up confidence and move forward with more research projects. The diary entry was a vivid description of how I thought of conducting research after the acceptance of one manuscript in the third year of my PhD.

*A day of achievement indeed. I was jogging when my friend told me excitedly that one manuscript was accepted. I felt so much relieved when I heard about it, and a lot of memories flashed back into my mind. It has been almost three years since I was enrolled in the PhD program, and it is such a difficult journey…I still remember the days and nights I collected and analyzed the data, the emotions I experienced in the process, the discussions with other researchers, etc. Learning to collect and analyze data, writing in a foreign language, and revising manuscripts, have all become important parts of my daily life…These experiences have enlightened my life with hope. I do not remember how many times we have discussed and revised the manuscripts. But I know that although sometimes I was exhausted, I felt empowered. My true feelings are that doing research is challenging but rewarding. I am happy to see what I have been doing in the past three years* (Diary, 14 August 2021).

### 4.3. Achieving personal growth

During the course of publication and dissertation writing, the anxiety I experienced brought me some sense of enjoyment, which led to better personal growth in various aspects, such as sustained engagement and improved autonomy. I not only stayed engaged in academic research, but also gradually had sustained engagement with research. Then I weaved new projects into my research. The research interest is driven by experiencing both anxiety and enjoyment, and retaining research interest through more research topics and a strong support network. In stressful situations, my reflections empowered me with the ability of better management, i.e., to mitigate different stresses, seek various social support, and build a research community to decrease tension from publication and dissertation writing. I could better position myself and have more willingness to seek knowledge and find ways to improve EFL writing skills. The growth helped me become a better version of myself.

#### 4.3.1. Sustained engagement

Sustained engagement was an important part of my personal growth during the PhD. It brought me a step closer to well-being, and consisted of cognitive engagement, emotional engagement, and research engagement. The cognitive engagement provided me with internal cognitive engagement and facilitated both the emotional engagement and research engagement. The emotional engagement I gained brought me abundant emotional resources to deal with the research demand, especially EFL writing. It further harnessed myself into research engagement. Such a combination of emotional engagement and research engagement improved my autonomy. Th diary entry described how I developed my research interest and engaged in doing research in the beginning of the second year of my PhD.

*1:20 p.m. in winter. It has been one year since I enrolled in the program…I could not believe I have so many obstacles. I should take a more self-distanced perspective to assess my writing ability. I seem to have better known myself, with more English writing experiences and emotional experiences. I have fallen in love with research design and data analysis. It has become an important part of my life. Anxiety or enjoyment, I feel pleasant to welcome both. I hope that I could make more progress in the future. Though there would be more difficulties and a new round of challenges, I have gained confidence* (4 January 2022).

#### 4.3.2. Improved autonomy

Enjoyment I gained from engagement facilitated the improvement of autonomy. I realized the importance of engaging in research in a more self-determined way, because I knew that this kind of autonomy helped generate better engagement, especially research engagement. The improved autonomy included self-regulation strategies and time management skills. Compared with my past self, I improved the self-regulation strategies and developed some expertise during the 4 years. Such self-regulation strategies were helpful for the improvement of emotion management and research scheduling. The diary entry depicted how I improved these two aspects in the second year of my PhD.

*I could complete the research writing task on a daily basis. There was boredom sometimes, and I felt a little bit lonely in the writing process. However, I could gradually adjust myself and focus more on it after regulating my emotions. I tried to be productive every single day. Also, I could get rid of the negative emotions through writing about my personal experiences, including the challenges and difficulties, the ways of how I calmed myself down through various ways, and the anguish I had in the writing and revision process…I gradually got autonomy satisfaction from writing and could better regulate my emotions. I like doing this in a self-determined way. It brings me a lot of fun* (Diary, 12 December 2020).

Improving the ability to communicate and interpret emotions is an important part of improved autonomy. In developing my research interest, I experienced both positive emotions (e.g., acceptance, satisfaction, and happiness) and negative emotions (e.g., frustration, anxiety, and disappointment). These mixed emotions were gradually alleviated through a deeper understanding of feedback from supervisors, reviewers, and editors. I could better comprehend their comments, with which I could surpass past experiences and set new challenging goals. I recorded how I interpreted my emotions in one diary entry.

*4:00 a.m. in the early morning. I have been working day and night these years, and finally some manuscripts were published. Up till now, four manuscripts have been accepted by SSCI-indexed journals. Then I have gained more confidence in academic writing as an EFL PhD student. Although I know there are still a lot of work to be done in the future, I have confidence in improving myself. I believe doing in my way can help me make improvements in using more idiomatic language, building up a more logical and coherent argument, etc.* (Diary, 16 September 2021).

Time management skills improved my work efficiency. Such improvement brought me more autonomy satisfaction and engagement. I wrote a diary entry before graduation, which reflected on my time management skills in the past 4 years.

*Time really flies. Soon I am going to graduate from this university. Reflecting on the past 4 years, I believe that good time management strategies are important for completing different tasks and maintaining positive emotions. A proper allocation of time is essential for completion of the work and managing the emotions that work brings to me. My time schedule is the regular workout in the morning, writing academic papers in the day time, enjoying family time after work, and then doing research. It has become a fixed daily pattern for me. Even if I had no idea of what to write, I still wrote diary entries to record my work experiences and emotional experiences. Then I could better reconcile with myself (Diary, 10 June 2022).*

During the 4 years from September 2018 to June 2022, I wrote 550 research diary entries. Looking back on my personal experiences described in the diary entries, I treasure the experiences as a PhD student, although there were a lot of anxiety. I also treasure diary writing, as it has become an emotional outlet for me. Anxiety, as part of my life, provided me a valuable opportunity to reflect on myself, and thus I could seek ways to face and manage anxiety, which led to a sense of enjoyment, personal growth, and well-being.

## 5. Discussion

The 4 years’ PhD trajectory brought forth both challenges and difficulties for me. The experiences are similar to those reported in previous studies (e.g., Park, 2005; Barry et al., 2018; Liu and Abliz, 2019; Huang, 2020; Beasy et al., 2021). The experiences covered both research and emotions, which echo with the call for attention to PhD students’ experiences, i.e., research experiences (e.g., Casanave, 2010, 2019; De Magalhaes et al., 2019) and emotional experiences (e.g., Aitchison et al., 2012; Cotterall, 2013; Russell-Pinson and Harris, 2019).

During the 4 years’ PhD program, I experienced complicated and varied emotions, and these emotions were part of a PhD student’s normal experiences (Casanave, 2014). Among these emotions, there were a large proportion of negative emotions, such as anguish, distress, and anxiety. I realized the harmfulness of the negative emotions (Russell-Pinson and Harris, 2019), the benefits of making use of positive emotions in providing motivational energy (McCormack, 2009), and the usefulness of research diary writing (Engin, 2011; Browne, 2013; Glass et al., 2019) in scaffolding my study and healing my emotions. Accordingly, I set up my mind and made great effort to compromise and negotiate with the ongoing negative emotions, especially anxiety, through writing research diaries and seeking sources of social support. This process of negotiating my negative internal dialog (Badenhorst, 2018) promoted sustained research engagement and improved my autonomy. Gradually I embraced a sense of enjoyment and achieved personal growth. This process was a trajectory of maintaining and enhancing PhD well-being.

I was fraught with anxiety during the PhD, including anxiety from publication and dissertation writing, foreign language writing anxiety, and anxiety from individual stressors. The findings of anxiety from publication and dissertation writing corroborate recent studies (e.g., Berg et al., 2016; Mura and Wijesinghe, 2022), which explored PhD students’ negative emotional experiences. Foreign language writing was the second source of anxiety. Such anxiety sometimes caused me to procrastinate my research, which are in line with the findings of some studies (e.g., Taso et al., 2017; Tahmouresi and Papi, 2021). However, I could manage my emotions and move forward with manuscript writing and revising, and gradually developed the ability to interpret and reconcile negative emotions. Individual stressors were the last sources causing anxiety. Different sources of anxiety raised my awareness of identity as a novice researcher. Realizing that I possessed most of the commonalities of a novice researcher eased and decreased my anxiety. As such, I made every effort to perceive myself in a more self-distanced perspective and renegotiate the state of anxiety. This helped reduce the negative mediation of anxiety. I wrote research diary entries to record and reflect on my research and emotional experiences, which helped soothe myself. Through actively engaging in writing diary entries, I realized that diaries could help further reveal my hidden and suppressed emotions and then release these emotions to a large extent. Since then, I viewed diary writing as a useful method (e.g., Alaszewski, 2006; Hyers, 2018) and a cathartic tool (Browne, 2013), and made full use of its role in scaffolding my research (Engin, 2011) and healing effects (Glass et al., 2019). Surprisingly, with time passing by, I perceived some positive changes that keeping diaries brought to me, and I inter-weaved diary writing with my PhD life. To cope with foreign language writing anxiety, I developed the ability to set up clearer arguments, find more authorized resources, and better express my voice with the scaffolding from my supervisor, reviewer comments, and other sources. To reduce anxiety from individual stressors, I tried to discuss with family members and colleagues. Such ways eased my anxiety and shifted my focus from being fraught with anxiety to a sense of enjoyment.

But I gained a sense of enjoyment during the PhD, including enjoyment from the support network and enjoyment from conducting research. I felt enjoyment from self-support through writing research diaries and social support through receiving constructive and positive comments. The findings of self-support corroborate previous studies (e.g., Engin, 2011; Browne, 2013), which stressed the role of research diary writing in helping PhD students heal in their fieldwork. Social support sources were various, but the findings showed how the feedback from the supervisor and reviewers helped me develop emotions from anxiety to enjoyment after managing the anxiety and focusing more on feedback or comments. This can be explained by the important role of supervisors in fostering doctoral satisfaction and degree completion (e.g., Barnes and Randall, 2012; McAlpine and McKinnon, 2013; Xu et al., 2021) and the usefulness of reviewer comments in the development of PhD students’ academic writing (e.g., Yu and Jiang, 2022). Enjoyment from conducting research included revision of manuscripts and acceptance of research articles. Such enjoyment was due to the co-development of my research engagement and EFL writing performance, and it could exert some positive influences on my cognitive processing styles and research-related behaviors, as indicated by the positive emotion theory (Fredrickson, 2001). Enjoyment, as a kind of positive emotion, helped me extend my research scope and interest after graduation, which would lead to my life-long personal growth.

I also gained personal growth in sustained engagement and improved autonomy. Personal growth, as a part of PhD students’ well-being (Ryff, 1989), led to an internal reflection on myself (Haynes et al., 2012), being true to myself (Schmidt and Umans, 2014), and self-care (Kumar and Cavallaro, 2018). My personal experiences illustrated the sustained engagement in finding research interest and engaging in more research projects. Such sustained engagement might motivate students to contribute more to the field (Beasy et al., 2021). The improved autonomy led me to a better productivity and efficiency in research and brought me opportunities of adhering to new expectations. Once fulfilling these expectations, I would have more emotional rewards, such as enjoyment, relief, and hope. All these emotions are conducive to my personal development and well-being. I thus enjoyed the trajectory more and regarded the four-year PhD experiences as a valuable part of my life journey.

## 6. Conclusion

This study has explored my personal experiences as a PhD student using an autoethnographic approach and revealed how I gained a sense of enjoyment and achieved personal growth after experiencing anxiety over a four-year period. Although these narratives were highly contextualized in my own personal experiences, it points to the common and wider experiences of PhD students. The analysis of my experiences enabled me to recognize the salient role of research diary writing in scaffolding PhD research and providing emotional support. I feel strongly that it is in this process that I could view all my emotions as agentive and embrace more enjoyment in a trajectory full of difficulties, challenges, and stress. Such enjoyment brought me a sense of relief, helped me avoid the harmful outcome of anxiety, and brought me back to sustained engagement.

Through analyzing research diary entries, I delineated my research experiences and complex emotional trajectories and took initiatives to seek self-support and social support of the kind that can maintain PhD students’ well-being. This study suggests that PhD students can and should develop their abilities to reduce anxiety and gain enjoyment. It is important that PhD students should have confidence in confronting the complex emotions and read related blog posts at a blog or website, which are shared by other PhD students. Such a way might help students alleviate anxiety and enhance well-being during their PhD. It is also necessary for PhD students to seek social support from supervisors, family, and institutions to deal with the ongoing negative emotions.

This study has contributed to the understanding of PhD students’ experiences through providing an insider’s perspective. PhD students are advised to cope with their negative emotions (i.e., anxiety and anguish) through diary writing and channel more positive emotions (i.e., enjoyment and relief) into their study. To help PhD students gain more sense of enjoyment during their PhD, supervisors can guide their students to reflect on their research and emotional experiences by incorporating research diary writing as a scaffolding tool. Such scaffolding opportunities would support students in the enhancement of well-being. Higher education institutions are suggested to use the low stakes research diary writing to help PhD students maintain and enhance their well-being.

This study has confirmed the usefulness of autoethnography as an empowering research method to explore PhD students’ experiences and make full use of the embodied experiences (Adams et al., 2014). Such an approach uses self-written research diaries as the data source and could help PhD students document personal experiences, engage in self-reflection, and improve emotion management and self-regulation strategies.

This study has two limitations. This study represented my own perspectives and explored my personal experience as an EFL PhD student across 4 years. It neither reached generalizable conclusions, nor suited all. Also, this study could have been done with the collaborative autoethnographic approach. Future research could use a collaborative approach to explore the personal experiences of more PhD students in different disciplines and contexts.

## Data availability statement

The raw data supporting the conclusions of this article will be made available by the authors, without undue reservation.

## Ethics statement

The studies involving humans were approved by the Research Ethics Board of Shandong University (reference no. ECSBMSSDU2018-1-051). The participant provided her written informed consent to participate in this study. Written informed consent was obtained from the individual(s) for the publication of any potentially identifiable images or data included in this article.

## Author contributions

The author confirms being the sole contributor of this work and has approved it for publication.

## Funding

This work was supported by China Scholarship Council (grant number 202102515001).

## Conflict of interest

The author declares that the research was conducted in the absence of any commercial or financial relationships that could be construed as a potential conflict of interest.

## Publisher’s note

All claims expressed in this article are solely those of the authors and do not necessarily represent those of their affiliated organizations, or those of the publisher, the editors and the reviewers. Any product that may be evaluated in this article, or claim that may be made by its manufacturer, is not guaranteed or endorsed by the publisher.
